# Improving the Secretion of a Methyl Parathion Hydrolase in *Pichia pastoris* by Modifying Its N-Terminal Sequence

**DOI:** 10.1371/journal.pone.0096974

**Published:** 2014-05-07

**Authors:** Ping Wang, Lu Huang, Hu Jiang, Jian Tian, Xiaoyu Chu, Ningfeng Wu

**Affiliations:** 1 Biotechnology Research Institute, Chinese Academy of Agricultural Sciences, Beijing, P. R. China; 2 Key Laboratory for Feed Biotechnology of the Ministry of Agriculture, Feed Research Institute, Chinese Academy of Agricultural Sciences, Beijing, P. R. China; Weizmann Institute of Science, Israel

## Abstract

*Pichia pastoris* is commonly used to express and secrete target proteins, although not all recombinant proteins can be successfully produced. In this study, we used methyl parathion hydrolase (MPH) from *Ochrobactrum* sp. M231 as a model to study the importance of the N-terminus of the protein for its secretion. While MPH can be efficiently expressed intracellularly in *P. pastoris*, it is not secreted into the extracellular environment. Three MPH mutants (N_66_-MPH, D_10_-MPH, and N_9_-MPH) were constructed through modification of its N-terminus, and the secretion of each by *P. pastoris* was improved when compared to wild-type MPH. The level of secreted D_10_-MPH was increased to 0.21 U/mL, while that of N_9_-MPH was enhanced to 0.16 U/mL. Although N_66_-MPH was not enzymatically active, it was secreted efficiently, and was identified by SDS-PAGE. These results demonstrate that the secretion of heterologous proteins in *P. pastoris* may be improved by modifying their N-terminal structures.

## Introduction

*Pichia pastoris* is a methylotrophic yeast that has been genetically engineered to express heterologous proteins for use in research, as well as industrial and pharmaceutical applications [1]. The use of *P. pastoris* as a host offers many advantages compared with other expression systems. For example, the yeast can be used to express large amounts of extracellular proteins at relatively little cost [2]. The preferential secretion of recombinant proteins allows for the direct isolation of target proteins from culture media, eliminating the need for high-cost, low-yield cell disruption. Furthermore, this feature limits the toxicity issues resulting from intracellular accumulation of target proteins [2]. In the *P. pastoris* expression system, signal peptides are commonly included in commercially available vectors, and so can be attached to the recombinant protein, causing it to be exported from the cell. Because *P. pastoris* secretes few intrinsic proteins, the recombinant protein forms the major polypeptide species found in the extracellular growth medium, which facilitates purification of the heterologous protein [3].

However, the *P. pastoris* system also has several limitations. Many recombinant proteins that should be secreted are retained inside the yeast cell [4]–[6]. Furthermore, these proteins are often mis-folded and degraded intracellularly, which can be a major problem [7]. Because the system is used widely, a number of strategies for enhancing protein production have been developed. However, most strategies do not target the protein itself, but instead modify other aspects of the process, such as the host [8], promoter [8], signal peptide [9], [10], chaperone [11], fusion proteins [12], protease [13], fermentation conditions [14]–[18], codon optimization [19], or gene copy number [20]. Although these methods can effectively enhance the expression of some proteins, it is challenging to enhance the secretion of certain proteins that are retained in the cell. Previous studies have shown that the rational design of the internal regions of a protein can enhance its secretion by *P. pastoris.* [21]–[24] Therefore, some secretion signals that affect protein secretion must exist in the internal regions of proteins. It may therefore be necessary to modify the sequence or structure of a protein to enhance its secretion.

In this study, we used methyl parathion hydrolase (MPH) from *Ochrobactrum* sp. M231 to investigate the effect of modification of its N-terminus on secretion. Organophosphorus hydrolases (OPHs) play important roles in the decontamination and bioremediation of environments polluted by organophosphate pesticides [25]–[33]. MPH, isolated by our lab from *Ochrobactrum* sp. M231 in 2008 [29], can efficiently and specifically degrade methyl parathion, but it cannot be secreted from *P. pastoris* [29], [33]–[35]. However, another OPH, OPHC2, isolated from *Pseudomonas pseudoalcaligenes*, also by our laboratory [32], has a similar three-dimensional structure to MPH. It has been over-expressed and efficiently secreted at concentrations of up to 5.5 g/L in *P. pastoris* [31], [32]. These two proteins have the same function and share a sequence identity of 47.7%. However, they have different secretion patterns when expressed in the same *P. pastoris* expression system with an identical promoter and signal peptide. Although it has been reported that integrating 12 copies of the MPH expression cassette into the *P. pastoris* system can significantly improve the secretion of MPH [36] and that MPH can be expressed and secreted by *Bacillus subtilis* WB800 [37], secretion levels with these two methods are still close to 100-fold lower than the levels of OPHC2 secreted by *P. pastoris*. We attempted to identify the protein sequence factor that affected the secretion of MPH by *P. pastoris*. Sequence and structural analyses revealed that MPH and OPHC2 have different N-terminal regions. Therefore, the aim of this study was to improve the secretion of MPH by *P. pastoris* through the modification of its N-terminal structure.

## Materials and Methods

### Strains, Plasmids, and Media

The GenBank accession numbers of *Ochrobactrum* sp. M231 MPH and OPHC2 are ACC63894 and CAE53631, respectively. *Escherichia coli* strain Top10 and the plasmid pGEM-T were purchased from Promega Corp. (Madison, WI, USA). *E. coli* strain BL21(DE3) and the expression plasmid pET-30a(+) were purchased from Novagen (Darmstadt, Germany). *P. pastoris* strain GS115 and the vector pPIC9 were purchased from Invitrogen (Carlsbad, CA).

*E. coli* cells were cultured aerobically at 37°C in Luria-Bertani medium. Minimal dextrose (MD) medium, buffered complex glycerol medium (BMGY), yeast extract peptone dextrose medium (YPD), and buffered complex menthol medium (BMMY) were prepared according to the manufacturer’s instructions (Invitrogen).

### The Design of MPH Mutants

The SCHEMA software [38] was used to compare recombination sites in the N-termini of MPH and OPHC2, using standard parameters, resulting in N-terminal blocks comprising 66 amino acids of MPH, and 68 amino acids of OPHC2. We designed a chimera (N_66_-MPH), where a 68-amino-acid block of OPHC2 was used to replace the 66 N-terminal amino acids of MPH. Wild-type MPH has a coil structure formed by 10 amino acids at its N-terminus, which might prevent the secretory pathway from recognizing its α-factor signal peptide. We therefore designed a deletion mutant (D_10_-MPH, in which the 10 N-terminal amino acids of MPH had been removed), and a chimera (N_9_-MPH, in which the 9 N-terminal amino acids of MPH were replaced by those from OPHC2).

### Construction of Expression Vectors in *E. coli*

Mutant vectors were synthesized using the plasmids pET-30a(+)-*mph* [33] and pET-30a(+)-*ophc2* [31], recombinant plasmids containing *mph* and *ophc2*, respectively, as the templates. Two plasmids (N_66_-MPH and N_9_-MPH) were then generated through homologous recombination, as described previously [35], using the primers listed in Table S1 in File S1. N_66_-MPH was generated from homologous recombination of three fragments: fragment 1 (using primers MPH-F-66 and pET30-R) and fragment 2 (using primers MPH-R-0 and pET30-F) were amplified from the pET-30a(+)-*mph* plasmid, while fragment 3 (using primers OPHC2-F-1 and OPHC2-R-68) was amplified from the pET-30a(+)-*ophc2* vector. N_9_-MPH was generated from homologous recombination of two fragments: fragment 1 (using primers N_9_ *-*R and pET30-F) and fragment 2 (using primers N_9_ *-*F and pET30-R) were amplified from the pET-30a(+)-*mph* plasmid. The *D*_10_ *-mph* gene was amplified from pET-30a(+)-*mph* with the primers D_10_-F and D_10_-R. The PCR products were digested with *Eco*RI and *Not*I, and cloned into pET-30a(+). To verify the inserted genes, DNA sequencing was performed at the State Key Laboratory of Crop Genetic Improvement, Chinese Academy of Agricultural Sciences (Beijing, China). The verified plasmids were transformed into competent *E. coli* BL21(DE3) cells for expression.

### Expression, Purification, and Quantification of Wild-type MPH and Mutants

A single colony of the transformed *E. coli* was cultured in Luria-Bertani liquid medium containing 50 µg/mL kanamycin (LB-kana) at 37°C overnight, and then inoculated to fresh LB-kana (1∶100 dilution) and incubated again at 37°C. When the OD_600_ of the culture reached 0.5, isopropyl β-D-1-thiogalactopyranoside (final concentration, 0.4 mM) was added. Cultures were incubated for an additional 18–20 h at 16°C. The cells were then collected by centrifugation and disrupted by sonication. The recombinant proteins were purified with Ni-NTA Superflow (QIAGEN, USA) according to the manufacturer’s instructions. The final concentration of the purified protein was determined using the Bio-Rad Protein Assay kit (Bio-Rad, Hercules, CA, USA).

### Enzymatic Properties of Wild-type and Mutant MPH

The standard enzyme assay and determination of kinetic parameters were performed as described by the reference [35]. The determination of the enzymatic properties of wild-type and mutant MPH was performed according to methods described previously [33], [34].

### Construction of Expression Vector and Transformation of P. pastoris

The wild-type and mutant plasmids were digested with *Eco*RV and *Not*I, and ligated into pPIC9. All plasmids were transformed into Top10 cells and their fidelity was confirmed by sequencing.

The plasmids containing wild-type and mutant vectors were extracted using a plasmid extraction kit (TIANGEN, Beijing, China). *Bgl*II was used to linearize 5–10-µg recombinant DNA, which was then transformed into *P. pastoris* GS115 cells using the Gene Pulser system (Bio-Rad; conditions used: 2.5 kV, 25 µF, and 400 Ω). His^+^ transformants were selected on MD plates, and the genomic DNA was extracted and analyzed by PCR using the primers 5′-AOX1 and 3′-AOX1 (Table S1 in File S1).

### Selection of High-producing Recombinant *P. pastoris* Strains

After transformation, the His^+^ transformants from the MD plates were grown in 3-mL BMGY, and induced in 1-mL BMMY for 48 h. Next, 100 clones each from the wild type and three mutant MPH transformants (400 clones in total) were assessed for the secretion of the expressed proteins using the standard enzyme assay. We also selected high-producing recombinant *P. pastoris* strains for the wild type and each of the three mutants for the shake-flask culture.

### Expression of MPH and Mutant Proteins in Shake-flask Culture

The colonies of His^+^ transformants exhibiting MPH activity were inoculated into 45-mL BMGY at 28°C with constant shaking at 200 rpm until the OD_600_ reached 5.0. Cells were pelleted by centrifugation and resuspended in 15-mL BMMY, then induced at 28°C with constant shaking at 200 rpm for 120 h. Methanol was added to a final concentration of 0.5% (v/v) every 24 h. The culture supernatant and cells were harvested by centrifugation (12,000 g, 3 min, 4°C) to analyze MPH activity according to methods described previously [36]. MPH activity in the supernatant and cells was determined using the standard enzyme assay.

### Isolation of Yeast RNA and Quantitative Real-time PCR

Yeast expression cultures were induced for 1 day with 0.5% methanol, and adjusted to an OD_600_ of 8.0. Cell pellets from 1-mL samples of the cultures were collected by centrifugation and lysed by grinding in liquid nitrogen. Total RNA was isolated using TRIzol (TIANGEN, Beijing, China) following the manufacturer’s instructions, and cDNA was synthesized using the TIANScript RT Kit (TIANGEN, Beijing, China). Duplicate PCR reactions were performed on an ABI PRISM 7700 sequence detection system (Applied Biosystems, Weiterstadt, Germany) with standard conditions (50°C for 2 min; 95°C for 10 min; and 45 cycles of 95°C for 15 sec, and 60°C for 1 min) using TransStart Probe qPCR SuperMix (Transgen, Beijing, China) following the manufacturer’s recommendations. The primers used for *mph* were mph-mF and mph-mR (Table S1 in File S1). Yeast *gapdh* was amplified as a control for normalization using the primers GAPDH-F and GAPDH-R (Table S1 in File S1). All primers were used at a final concentration of 0.2 µM.

### Gene Copy Number Determination by Real-time PCR

Genomic DNA was prepared using the TIANamp Yeast DNA Kit (TIANGEN, Beijing, China). Gene copy numbers of *mph* and mutants were determined according to the absolute quantification method described by the reference [39], using the SYBR Green Real-time PCR Master Mix-Plus (Toyobo, Osaka, Japan). The primers and quantitative real-time PCR protocol were as described in the previous section.

### Protein Structure Analysis

The tertiary structures of MPH, OPHC2, and N_9_-MPH were constructed using the Discovery Studio software v.2.5.5 (Accelrys Software Inc., USA). Molecular dynamics simulations (MDS) of the protein structures were carried out for 10 ns at 300 K using the Gromacs v.4.5.5 software, as described previously [33]. The interaction energy was calculated using the VMD v.1.8.6 software, following standard protocols.

## Results

### Three-dimensional Models of MPH, OPHC2, and N_9_-MPH

**Figure 1:**
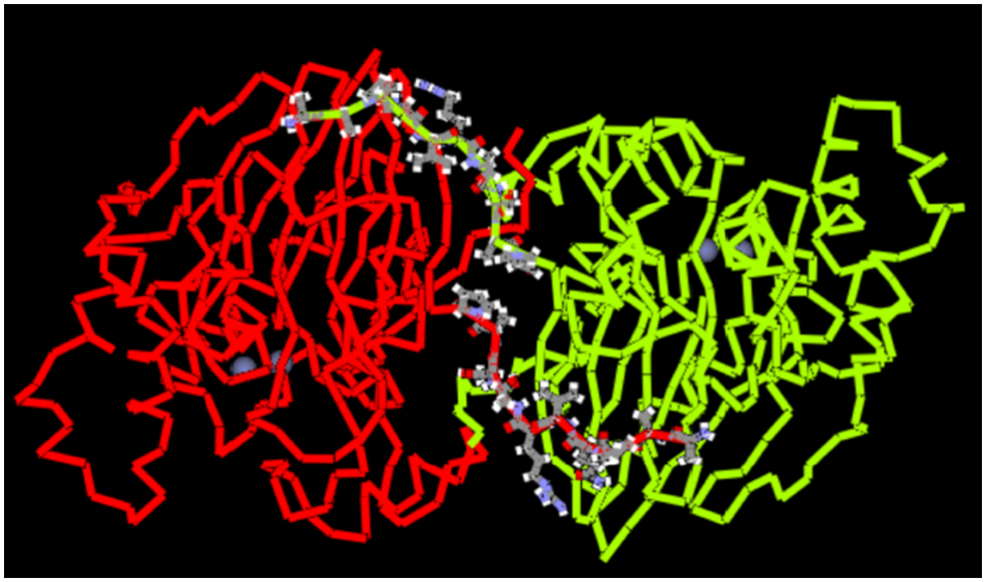
The 3D structure of MPH. The red and green chains are chains A and B of MPH, respectively. The 10 residues at the N-terminus are represented in ball and stick form. The zinc ion is shown as a gray ball.

The 3D structures of MPH, OPHC2, and N_9_-MPH were modeled based on the crystal structure of *Pseudomonas* sp. MPH (PDB reference: 1P9E) [40]. The resulting model (Figure 1) is a dimer that can be described as an αβ/βα sandwich, which is typical of metallo-hydrolase/oxidoreductase folding. As expected, the structures of MPH and OPHC2 were similar, since they have high sequence identity. Based on the models of the 3D protein structures, the recombination sites at the N-terminus were identified as residue 66 of MPH and 68 of OPHC2 using the Schema software [38].

We next assessed the importance of the N-terminus for protein secretion. As shown in Figure S1 in File S1, the nine residues at the N-terminus of MPH and OPHC2 are different, and only three residues are consistent when the sequences are aligned. However, the total sequence identity between these regions of MPH and OPHC2 is 47.7%. In addition, wild-type MPH has a coil structure in the first 10 amino acids of its N-terminus, which might prevent recognition of its α-factor signal peptide by the secretory pathway. Therefore, two MPH mutants (D_10_-MPH and N_9_-MPH) were constructed to assess the effect of these residues on the secretion of MPH. In D_10_-MPH, the 10 amino acids from the N-terminus were deleted, while in N_9_-MPH, the 9 N-terminal amino acids of MPH were replaced by those from OPHC2.

### Expression of MPH and Mutant Proteins in P. pastoris

**Figure 2:**
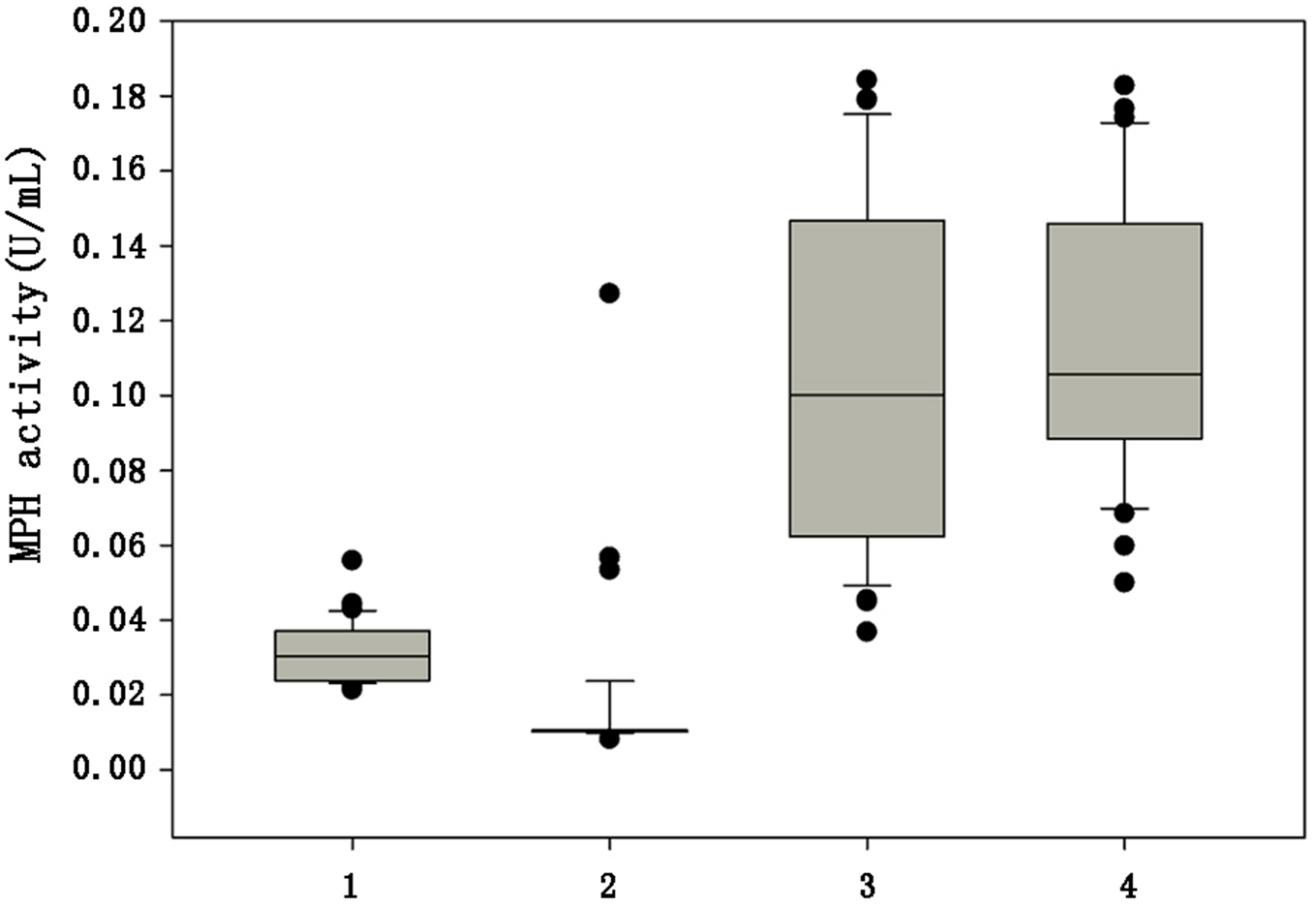
A box-whisker plot of enzyme activity in the culture supernatants of *P. pastoris* transformants . Boxes denote the interquartile range (IQR) between the first and third quartiles, while the line inside the box denotes the median. Whiskers denote the lowest and highest values within 1.5-fold the IQR from the first and third quartiles, while circles denote outliers. 1 = *P. pastoris* clones that expressed MPH, 2 = N_66_-MPH, 3 = D_10_-MPH, and 4 = N_9_-MPH.

To investigate the effects of the mutations on the expression and secretion of MPH in *P. pastoris*, pPIC9-based yeast expression constructs (pPIC9-*N_66_-mph*, pPIC9-*D_10_-mph*, pPIC9-*N_9_-mph*) were generated. One hundred clones each from wild-type and three mutant MPH transformants were grown, and the secretion of the expressed proteins was assessed using the standard enzyme assay. As shown in Figure 2, the supernatants from most D_10_-MPH and N_9_-MPH transformants showed MPH activity higher than 0.1 U. In contrast, supernatant from wild-type MPH transformants exhibited activity at 0.03 U. The N_66_-MPH clone, which had 34 mutated amino acids compared with wild-type MPH, had low enzymatic activity, even though SDS-PAGE confirmed its expression and secretion by *P. pastoris* (data not shown). We also selected the following high-producing recombinant *P. pastoris* strains of each gene for the next shake-flask culture: MPH-24# (0.03 U/mL), N_66_-MPH-5# (0.02 U/mL), D_10_-MPH-56# (0.15 U/mL), and N_9_-MPH-70# (0.15 U/mL).

### Production and Activity of Wild-type and Mutant MPH Proteins

**Figure 3:**
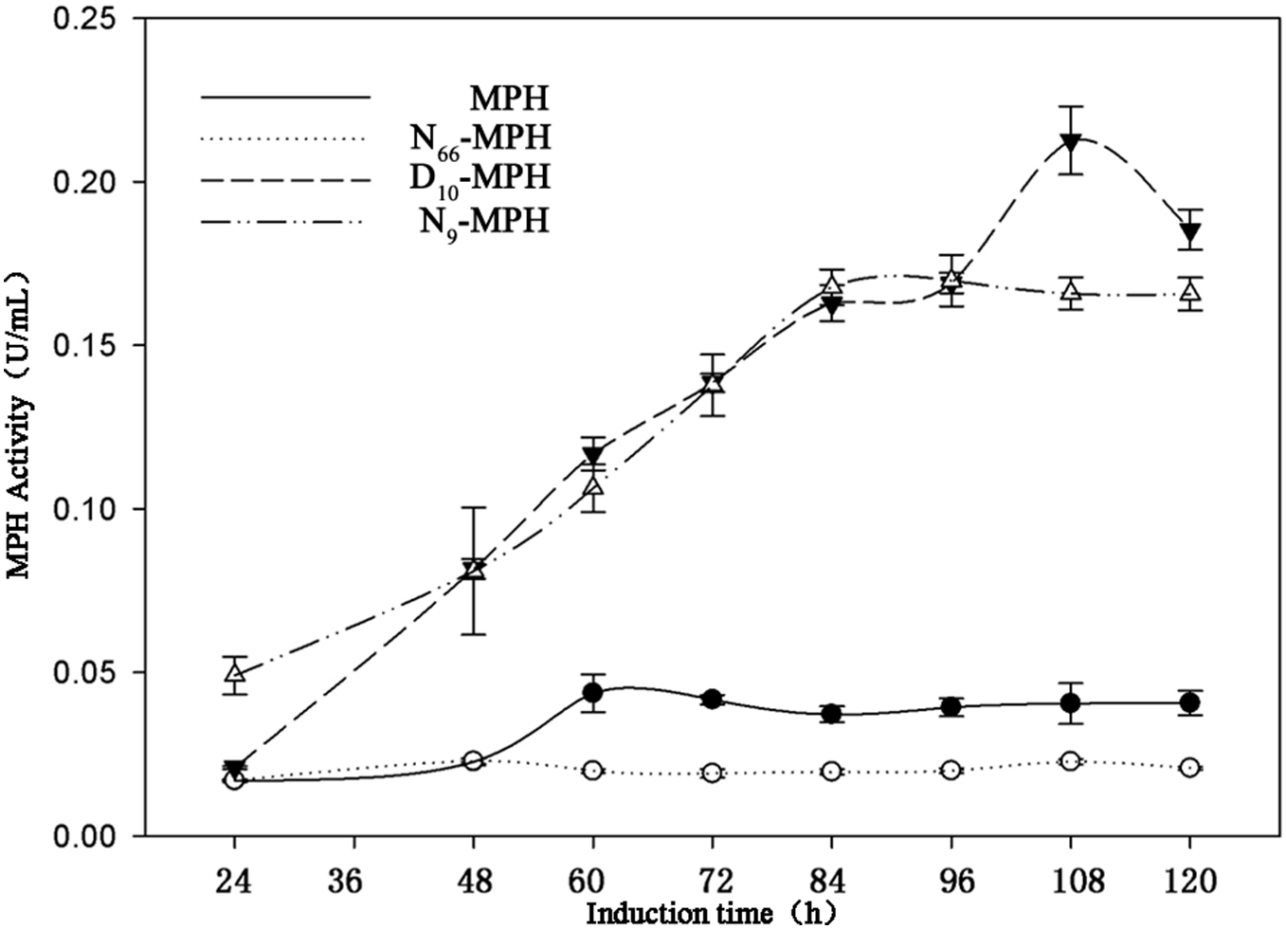
Enzyme activity in culture supernatants. MPH-24# (solid circle), N_66_-MPH-5# (hollow circle), D_10_-MPH-56# (solid triangle), and N_9_-MPH-70# (hollow triangle) were induced by methanol for various time periods (as indicated on the x-axis). The MPH activities in the supernatants were determined using standard enzyme assays and are shown on the y-axis. Enzyme activity is expressed as the mean of three samples, and error bars indicate standard deviation (SD).

**Figure 4:**
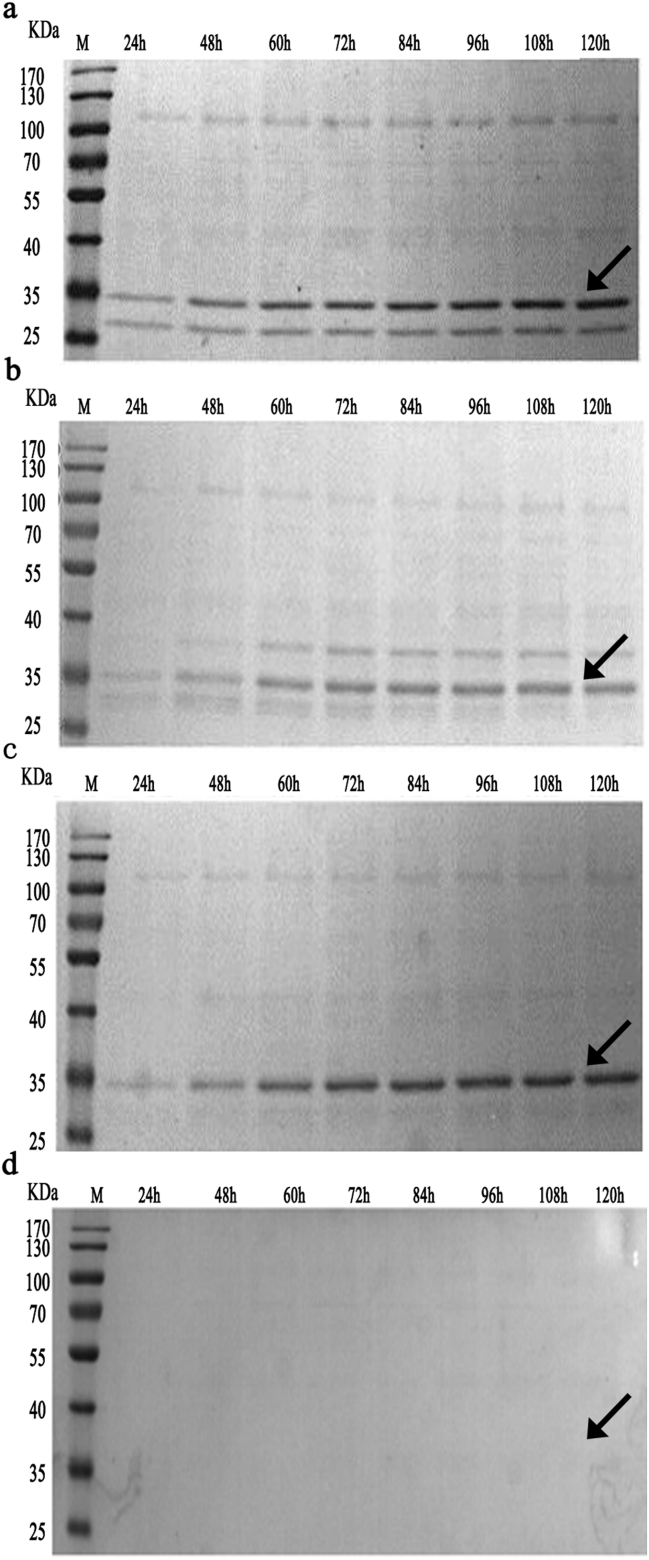
SDS-PAGE analysis of culture supernatants. Transformants were induced by methanol as indicated, and the supernatants were analyzed by SDS-PAGE. Blots show the supernatants of the a) N_66_-MPH-5#, b) D_10_-MPH-56#, c) N_9_-MPH-70#, and d) MPH-24# clones. Arrows indicate the positions of the bands corresponding to expressed proteins. Lane 1, standard protein molecular weight marker; lanes 2–9, culture supernatants after methanol induction for 24, 48, 60, 72, 84, 96, 108, or 120 h, respectively.

The four transformants (MPH-24#, N_66_-MPH-5#, D_10_-MPH-56#, and N_9_-MPH-70#) were cultured and the MPH activities in methanol-induced cultures at 28°C were calculated. As shown in Figure 3, the maximum activity was observed after 108 h for D_10_-MPH-56#, and 84 h for N_9_-MPH-70#. The maximum extracellular MPH activities of D_10_-MPH-56# and N_9_-MPH-70# cultures were 0.21 and 0.16 U/mL, respectively. The maximum activities of both the D_10_-MPH-56# and N_9_-MPH-70# supernatants were higher than that of MPH-24# (0.05 U/mL), while N_66_-MPH-5# showed the lowest extracellular MPH activity. The proteins in each supernatant were collected after 120 h of induction and analyzed by SDS-PAGE. As shown in Figure 4, N_66_-MPH-5#, D_10_-MPH-56#, and N_9_-MPH-70# all showed distinctive bands with a molecular weight of ∼35 kDa. The bands were subsequently identified as the appropriate MPHs by mass spectroscopy (data not shown). However, no band corresponding to MPH-24# was observed. These data suggest that N_66_-MPH, D_10_-MPH, and N_9_-MPH, but not MPH-24#, can be efficiently secreted from yeast cells. We also detected the intracellular MPH activity. As shown in Figure S2 in File S1, the strain containing wild-type MPH had higher intracellular MPH activity than the three mutant strains (N_9_-MPH, D_10_-MPH, and N_66_-MPH). Specifically, the intracellular MPH activity of *P. pastoris* strain MPH-24# reached 1.1 U/mL after 24 h, and eventually increased to 3 U/mL after 120 h (Figure S2b in File S1). These results indicate that the mutations facilitated secretion of the expressed proteins from the cell. Meanwhile, intracellular proteins are released upon cell lysis, which may explain why we did not detect MPH activity in supernatants from the initial cultures of wild-type MPH, but later identified weak MPH activity (Figure 3, Figure S2 in File S1). These experiments were repeated three times with similar results.

During the 5-day induction period, the four selected transformants had comparable growth rates (Figure S3 in File S1). This suggests that the increased MPH levels produced by the three mutants were not due to higher cell densities or enhanced proliferation.

### MPH mRNA Levels and Gene Copy Numbers in P. pastoris Transformants

**Figure 5:**
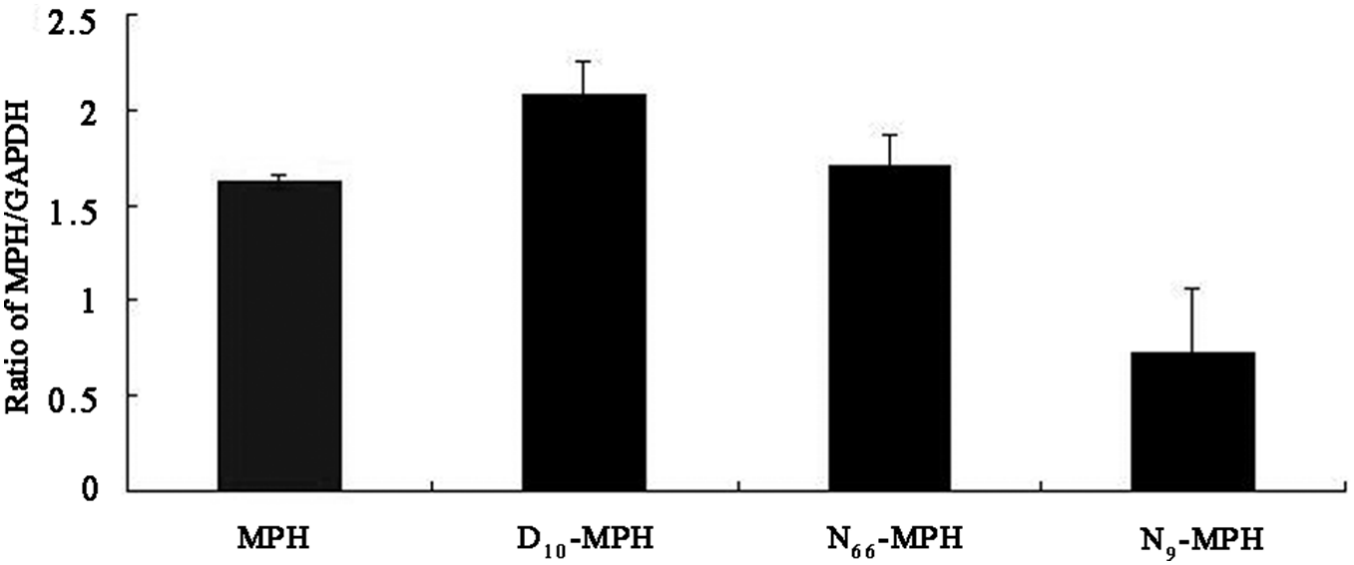
Analysis of *mph* mRNA levels. The expression of the recombinant cDNAs was assessed by quantitative real-time PCR, using GAPDH for normalization. Data are expressed as the means of duplicate samples, and error bars indicate standard deviation (SD).

We analyzed the mRNA levels expressed from the MPH-24#, N_66_-MPH-5#, D_10_-MPH-56#, and N_9_-MPH-70# vectors to determine whether differences in transcription contributed to the differential protein expression. After induction with methanol for 24 h, quantitative real-time PCR revealed that mRNA expression was highest for D_10_-MPH-56#. D_10_-MPH-56#, N_66_-MPH-5#, and N_9_-MPH-70# were expressed at 128%, 105%, and 45%, respectively, of the level of MPH-24# (Figure 5). These relatively small changes in mRNA expression clearly did not lead to the differences in protein secretion, as MPH-24# exhibited the lowest level of secreted protein. In addition, the data showed that the four *P. pastoris* transformants (MPH, N_66_-MPH, D_10_-MPH and N_9_-MPH) were single-copy clones (Table 1). Therefore, the differences in protein secretion were likely regulated at the post-transcriptional level.

**Table 1:** The copy number calculation of WT and mutant MPH according to absolute (Abs. Q).

Pichia pastoris strain	Abs. Q (SYBR Green)	Resulting copy number
N_66_-MPH-5#	1.1±0.1	1
D_10_-MPH-56#	1.3±0.1	1
N_9_-MPH-70#	0.8±0.1	1
WT MPH-24#	1.0±0.1	1

### Characterization of the Kinetics of Wild-type and Mutant Enzymes

The genes encoding MPH, N_66_-MPH, D_10_-MPH, and N_9_-MPH were cloned and expressed in *E. coli.* After purification, each expressed protein migrated as a single band with an approximate molecular mass of 38 kDa on SDS-PAGE (Figure S4 in File S1). We employed the *E. coli* system to express the wild-type and mutant proteins, as wild-type MPH was not secreted from *P. pastoris*. To perform an equal comparison, all four proteins were expressed in the *E. coli* expression system and the enzymatic activities were measured. The kinetic parameters of the enzymes are shown in Table 2. All of the mutants, except N_66_-MPH, had similar MPH activities to that of the wild-type enzyme. However, MPH exhibited 3.9- and 2.4-fold increased catalytic efficiency (*k_cat_/K_m_*) compared with D_10_-MPH and N_9_-MPH, respectively. The protein stability properties were also determined, as shown in Table S2 in File S1. The results indicate that the mutations did not significantly improve the protein stability properties, as their optimal pH values and temperatures were similar to those of the wild type. Therefore, the enhanced secretion of the mutant proteins was not a result of increased protein stability. These results indicate that the N-terminus of the protein is linked to the secretion of MPH from *P. pastoris*.

**Table 2:** The kinetic parameters of WT and mutant MPH.

	*k*_cat_ (min^−1^)	*K*_m_ (µM)	*k*_cat_/*K*_m_ (µM^−1 ^min^−1^)
WT MPH	253.80±11.41	73.17±4.70	3.47
D_10_-MPH^a^	77.53±2.66	86.87±4.01	0.89
N_9_-MPH^b^	208.80±9.14	145.5±6.51	1.44

a The first 10 amino acids of the N-terminus of MPH were deleted.

b The first nine amino acids from the N-terminus of MPH were replaced by those from OPHC2.

## Discussion

In this study, MPH from *Ochrobactrum* sp. M231 was selected and the importance of its N-terminus in its secretion by *P. pastoris* was determined. We swapped the corresponding block of sequence from OPHC2 based the Schema software analysis [38] and removed the N-terminal block of MPH according to the 3D protein structures. Our results revealed that the N-terminal region plays an important role in secretion. In addition, the improved secretion of the MPH mutants was not due to differences in growth rate, mRNA expression, gene copy number, or stability. Although the mutant proteins had reduced catalytic efficiency, the secretion of both D_10_-MPH and N_9_-MPH was improved significantly compared to wild-type MPH, as demonstrated by SDS-PAGE (Figure S5 in File S1).

Because the N-terminus of the protein plays an important role in its secretion, we used MDS to predict structural differences between the proteins. As shown in Figure 1, the N-terminus of each chain (A or B) directly interacts with the other chain, and thus plays an important role in dimer formation. The total interaction energy between chains A and B was calculated by MDS modeling (Figure S6 in File S1). MPH and OPHC2 have similar interaction energies between the chains. Specifically, the average interaction energies between chains A and B of MPH, OPHC2, and N_9_-MPH during the final 5 ns of MDS were –858.3, –847.3, and –627.1 kcal/mol, respectively. However, the average interaction energies of the N-terminus (the first 10 amino acids) and other regions of the protein for the final 5 ns of MDS were –670.0 and –530.9 kcal/mol for MPH and OPHC2, respectively. These results suggest that the N-terminus of OPHC2 was more flexible than that of MPH. As shown in Figure S6 in File S1, the interaction energies of the N-terminus of N_9_-MPH with the other protein regions was also lower than that of MPH. A more flexible N-terminus is therefore important for optimal protein secretion.

These results indicate that the N-terminus of the MPH protein contains a key sequence factor that affects its secretion from *P. pastoris*. In this study, we used a specific method to modify the protein N-terminal sequence. However, highly specific strategies will be necessary to optimize the N-terminal sequences of other proteins, and software should be developed for such work in the future.

## Supporting Information

File S1 **Supporting figures and tables.** This file contains Table S1-Table S2 and Figure S1-Figure S6. Table S1, The primers that involved in the construction of the mutants. Table S2, The enzymatic properties of WT and mutant MPH. Figure S1, The sequence alignment of N-terminal of the three proteins. Figure S2, Enzyme activity in culture supernatants (a) and cells (b). Figure S3, The growth kinetics of the selected transformants. Figure S4, SDS/PAGE analysis of the purified WT MPH and mutants (N66-MPH, D10-MPH, N9-MPH). Figure S5, SDS-PAGE analysis of culture supernatants from 72 hours methanol induction. Figure S6, The interaction energy of the protein OPCH2, MPH and N9-MPH.
(ZIP)
