# Supplementary material for: Improving the Secretion of a Methyl Parathion Hydrolase in Pichia pastoris by Modifying Its N-Terminal Sequence
Source: PLoS One. 2014 May 7;9(5):e96974. doi: 10.1371/journal.pone.0096974 (PMC4013123; doi:10.1371/journal.pone.0096974)
Supplement: File S1 — Supporting figures and tables. This file contains Table S1-Table S2 and Figure S1-Figure S6. Table S1, The primers that involved in the construction of the mutants. Table S2, The enzymatic properties of WT and mutant MPH. Figure S1, The sequence alignment of N-terminal of the three proteins. Figure S2, Enzyme activity in culture supernatants (a) and cells (b). Figure S3, The growth kinetics of the selected transformants. Figure S4, SDS/PAGE analysis of the purified WT MPH and mutants (N66-MPH, D10-MPH, N9-MPH). Figure S5, SDS-PAGE analysis of culture supernatants from 72 hours methanol induction. Figure S6, The interaction energy of the protein OPCH2, MPH and N9-MPH. (ZIP) [file pone.0096974.s001.zip › File1/Figure S2 in File S1.docx]

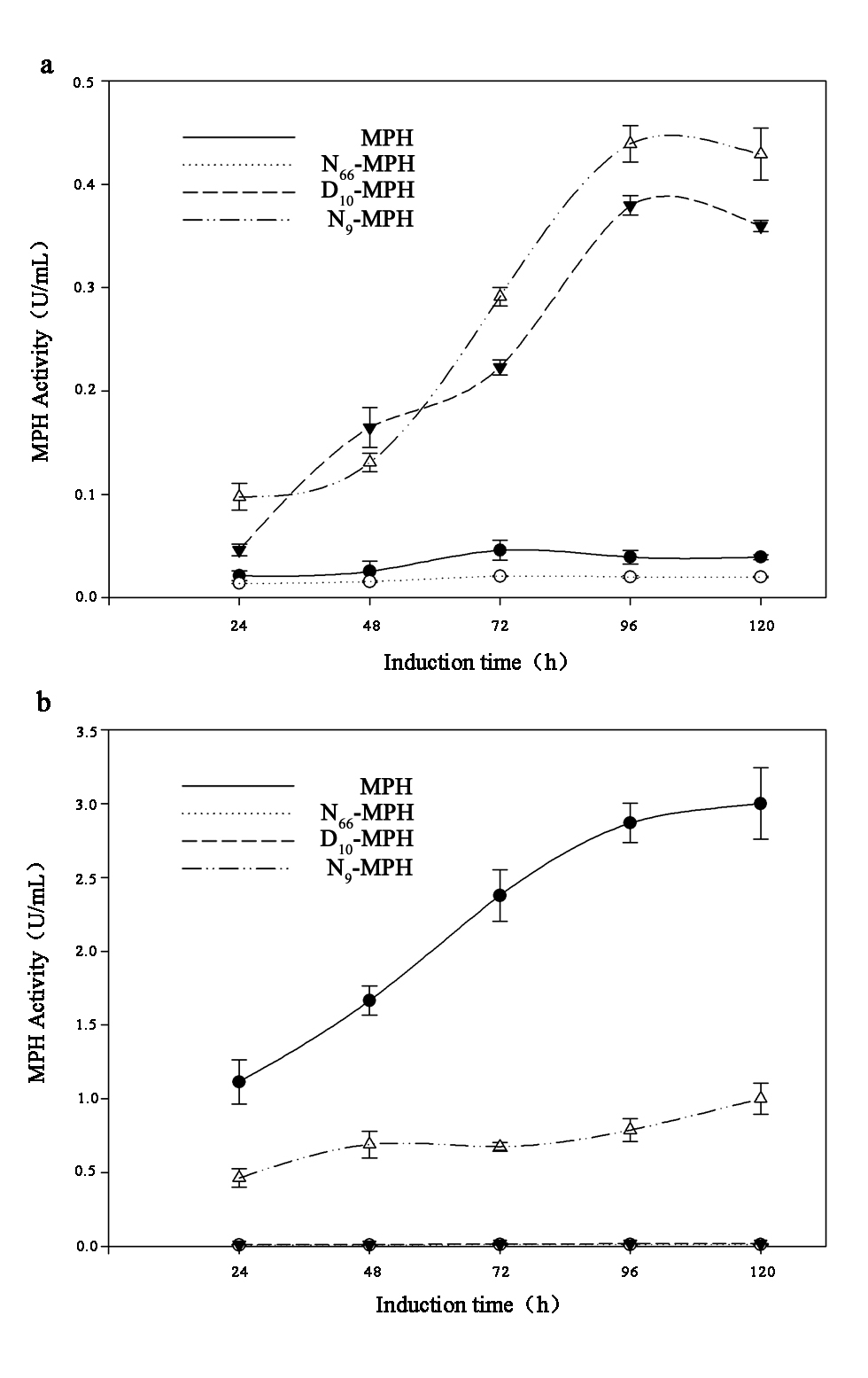


**Figure S2** **Enzyme activity in culture supernatants (a) and cells (b).** MPH-24#(solid circle), N_66_-MPH-5#(hollow circle), D_10_-MPH-56#(solid triangle), and N_9_-MPH-70#(hollow triangle) were induced by methanol for various times (as indicated as x-axis), and MPH activity in the supernatants and cell were determined using standard enzyme assays(as indicated as y-axis),.
