# Supplementary material for: Improving the Secretion of a Methyl Parathion Hydrolase in Pichia pastoris by Modifying Its N-Terminal Sequence
Source: PLoS One. 2014 May 7;9(5):e96974. doi: 10.1371/journal.pone.0096974 (PMC4013123; doi:10.1371/journal.pone.0096974)
Supplement: File S1 — Supporting figures and tables. This file contains Table S1-Table S2 and Figure S1-Figure S6. Table S1, The primers that involved in the construction of the mutants. Table S2, The enzymatic properties of WT and mutant MPH. Figure S1, The sequence alignment of N-terminal of the three proteins. Figure S2, Enzyme activity in culture supernatants (a) and cells (b). Figure S3, The growth kinetics of the selected transformants. Figure S4, SDS/PAGE analysis of the purified WT MPH and mutants (N66-MPH, D10-MPH, N9-MPH). Figure S5, SDS-PAGE analysis of culture supernatants from 72 hours methanol induction. Figure S6, The interaction energy of the protein OPCH2, MPH and N9-MPH. (ZIP) [file pone.0096974.s001.zip › File1/Figure S3 in File S1.docx]

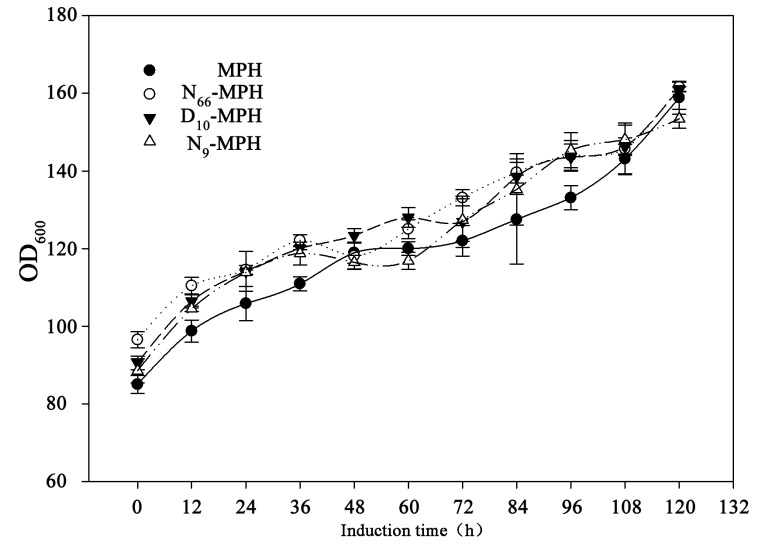


**Figure S3 The growth kinetics of the selected transformants.** MPH-24#, N_66_-MPH-5#, D_10_-MPH-56#, and N_9_-MPH-70# strain were inoculated in 45 mL BMGY at 28 °C with constant shaking at 200 rpm until the optical density at 600 nm (OD_600_) reached 5. The cell lysate were resuspended in 15 mL BMMY and induction performed at 28 °C, with constant shaking at 200 rpm for 120 h. Methanol was added to a final concentration of 0.5% (v/v) every 24 h. The optical density at 600 nm (OD_600_) were determined after induction.
