# Supplementary material for: Improving the Secretion of a Methyl Parathion Hydrolase in Pichia pastoris by Modifying Its N-Terminal Sequence
Source: PLoS One. 2014 May 7;9(5):e96974. doi: 10.1371/journal.pone.0096974 (PMC4013123; doi:10.1371/journal.pone.0096974)
Supplement: File S1 — Supporting figures and tables. This file contains Table S1-Table S2 and Figure S1-Figure S6. Table S1, The primers that involved in the construction of the mutants. Table S2, The enzymatic properties of WT and mutant MPH. Figure S1, The sequence alignment of N-terminal of the three proteins. Figure S2, Enzyme activity in culture supernatants (a) and cells (b). Figure S3, The growth kinetics of the selected transformants. Figure S4, SDS/PAGE analysis of the purified WT MPH and mutants (N66-MPH, D10-MPH, N9-MPH). Figure S5, SDS-PAGE analysis of culture supernatants from 72 hours methanol induction. Figure S6, The interaction energy of the protein OPCH2, MPH and N9-MPH. (ZIP) [file pone.0096974.s001.zip › File1/Table S1 in File S1.docx]

**Table S1** The primers that involved in the construction of the mutants

| primers | sequences |
| --- | --- |
| N_66_-F | GGATACTACAGAATGTTGCTGGGTGAC |
| N_66_-R | TTACTTTGGGTTAACGACGGAGTAGTT |
| OPHC2- F-1 | GCACCAGCACAACAGAAGACC |
| OPHC2- R-68 | CAAGTAAGCGTTGACAGCAGT |
| MPH - F-66 | GTCAACGCTTACTTGGTCAACACTGGTTCCAAGTTG |
| MPH - R-0 | CTGTTGTGCTGGTGCGATATCGCTGTCCATGTGCTGGCGTTC |
| pET30-F | TTACTGATGATGAACATGCCC |
| N_9_*-* R | GCACCAGCACAACAGAAGACCCAAGTTCCAGGATACTACAGAATG |
| pET30-R | GTTCATCATCAGTAACCCGTA |
| N_9_*-* F | AACTTGGGTCTTCTGTTGTGCTGGTGCGAATTCGGATCCGATATC |
| D_10_-F  D_10_-R | CCG**GAATTC**GGATACTACAGAATGTTGCTGGGT  ATTT**GCGGCCGC**TTACTTTGGGTTAACGACGGAGTA |
| 5’-AOX1 | GACTGGTTCCAATTGACAAGC |
| 3’-AOX1 | GCAAATGGCATTCTGACATCC |
| mph-mF | ATGGTTGGTGAGCAATTGGCTT |
| mph-mR | CCCGAGAAAGGCTTGAACTTAC |
| GAPDH-F | CAAGGATGCTCCAATGTTCGTT |
| GAPDH-R | CGGTCTTTTGAGTGGCGGTCAT |

The restriction sites were indicated by the underlined, bold sequences.

F: Forward primer. R: Reverse primer.
