# Supplementary material for: Improving the Secretion of a Methyl Parathion Hydrolase in Pichia pastoris by Modifying Its N-Terminal Sequence
Source: PLoS One. 2014 May 7;9(5):e96974. doi: 10.1371/journal.pone.0096974 (PMC4013123; doi:10.1371/journal.pone.0096974)
Supplement: File S1 — Supporting figures and tables. This file contains Table S1-Table S2 and Figure S1-Figure S6. Table S1, The primers that involved in the construction of the mutants. Table S2, The enzymatic properties of WT and mutant MPH. Figure S1, The sequence alignment of N-terminal of the three proteins. Figure S2, Enzyme activity in culture supernatants (a) and cells (b). Figure S3, The growth kinetics of the selected transformants. Figure S4, SDS/PAGE analysis of the purified WT MPH and mutants (N66-MPH, D10-MPH, N9-MPH). Figure S5, SDS-PAGE analysis of culture supernatants from 72 hours methanol induction. Figure S6, The interaction energy of the protein OPCH2, MPH and N9-MPH. (ZIP) [file pone.0096974.s001.zip › File1/Table S2 in File S1.docx]

**Table S2** The enzymatic properties of WT and mutant MPH.

|  | T_opt_ (℃) | t_50_ (℃) | optimum pH^a^ | t_1/2_ (min)^b^  (pH=6) |
| --- | --- | --- | --- | --- |
| WT MPH | 37 ± 0.5 | 62.1± 0.2 | 8 | 68 ± 6 |
| D_10_-MPH | 35 ± 1.0 | 60.9± 0.4 | 8 | 50 ± 3 |
| N_9_- MPH | 37 ± 0.5 | 63.3± 0.2 | 8 | 56 ± 2 |

a The optimum pH for MPH activity

b The half-lives of WT and its mutants at pH 6.0
